# Trichiniasis

**Published:** 1866-04

**Authors:** 


*Trichiniasis.*—This parasitic invasion of the human body is giving some uneasiness to the people of the North West, in various parts of the country. *Trichina-Spiralis* is the name given a parasite which penetrates the soft tissues of the body, particularly the voluntary muscles, and when introduced, gives rise to a train of symptoms most distressing and dangerous. The name which heads this article denotes that disease or condition produced by the ravages of the entozoa. Their introduction, it is supposed, occurs by the use of the flesh of animals as food, in which the trichinæ exist; passing into the bowels, they make their way into the muscles.

The following case is taken from the Detroit *Medical Review*, as reported for that journal by Herman Keifer, of Detroit Michigan:

"On the second of January last, I was called to see Mrs. M., a young woman of twenty-one years of age, who had been in this country about four months, having left Germany in June of last year. I found her in bed, having been ill for a week, unable to move herself in any way, from severe pain and soreness in all the muscles of her body. Pain in the throat almost wholly prevented swallowing, and opening the mouth sufficiently to show the tongue. Examination of her mouth and throat showed the tongue moist, a little coated, the uluva and tonsils natural. She had no appetite, but intense thirst, some nausea, bowels constipated; no particular pain in any part of the abdomen, and no tympanistis; sounds of heart and lungs normal, pulse 102, skin moist, perspiring easily; kidneys acting freely, the urine acid, pale yellow, containing no albumen, and without sediment; secretion of milk (she was nursing a child ten weeks old) diminished, no menses; mind undisturbed, but unable to sleep at all. Extreme sensitiveness prevailed in all parts of her body, to such a degree that simply holding her hand to examine the pulse caused intense pain. The case lingered as above for three weeks, without any material change, except increasing weakness, pulse more frequent, rising to 120 per minute, pains, thirst and sleeplessness more distressing. After sixteen days anasarca and profuse diaphoresis appeared; the œdema more marked in the lower extremities and constantly increasing. Diarrhœa set in, and an eruption of small red pimples (like that of variola on the second day of its appearance) spreading over the thorax, and she sank rapidly. On the 19th her mind became disturbed, and on the 21st day after my first visit she died.

As to the diagnosis of the case during the life of the patient, I confess I was somewhat puzzled during the first week I saw her. My first idea that it was an acute muscular rheumatism, soon had to be abandoned, as also the next one that it was typhoid fever, by the absence of the most important symptoms due to the diseases. In thinking over the matter again and again, I remembered a description of a case of Trichiniasis by Professor Friedrich, of Heidleburg, published in Lath's Medical Almanac for 1864. By a careful perusal of the case, I found such a coincidence of symptoms in my own, that I had from the moment not a doubt that the case was one of trichiniasis. Particularly striking were the pains in the throat, (by emigration of the trichina into the muscles of the pharynx) the intense thirst, the profuse sweating and anasarca, the restlessness and muscular sensitiveness. A microscopic examination of portions of the muscles of the abdomen and leg showed the presence of *trichina spiralis*. The portion from the abdomen was literally swarming with them, but in the leg they were more scattered.

As this disease is causing great alarm in Germany during the last few years, and as already several cases are reported as occurring in this country, (within a few weeks one died in Chataque Co., N. Y., under the care Dr. Krompein, and one or two are reported from Marietta Co., O., by Dr. Dingler,) there seems no reason why the disease should not spread to some extent here.

In the next number of the Review I shall present a synopsis of all that has come to my knowledge in regard to the *trichina*, with drawings from nature of the parasite in its various stages of development.

This case is reported in the hope that my experience may lead my brothers of the medical profession to keep a sharp lookout for this new enemy of the human race."

